# Evolution of Network Structure and Mechanical Properties in Autonomous-Strengthening Dental Adhesive

**DOI:** 10.3390/polym12092076

**Published:** 2020-09-12

**Authors:** Rizacan Sarikaya, Linyong Song, Qiang Ye, Anil Misra, Candan Tamerler, Paulette Spencer

**Affiliations:** 1 Institute for Bioengineering Research, University of Kansas, 1530 W. 15th Street, Lawrence, KS 66045-7609, USA; rsarikaya@ku.edu (R.S.); leonsong@ku.edu (L.S.); amisra@ku.edu (A.M.); ctamerler@ku.edu (C.T.); 2 Department of Mechanical Engineering, University of Kansas, 1530 W. 15th Street, Lawrence, KS 66045-7609, USA; 3 Department of Civil Engineering, University of Kansas, 1530 W. 15th Street, Lawrence, KS 66045-7609, USA; 4 Bioengineering Program, University of Kansas, 1530 W. 15th Street, Lawrence, KS 66045-7609, USA

**Keywords:** polymers, dental adhesive, autonomous strengthening, stress relaxation, mechanical property evolution, Prony series

## Abstract

The inherent degradation property of most dental resins in the mouth leads to the long-term release of degradation by-products at the adhesive/tooth interface. The by-products increase the virulence of cariogenic bacteria, provoking a degradative positive-feedback loop that leads to physicochemical and mechanical failure. Photoinduced free-radical polymerization and sol‒gel reactions have been coupled to produce a novel autonomous-strengthening adhesive with enhanced hydrolytic stability. This paper investigates the effect of network structure on time-dependent mechanical properties in adhesives with and without autonomous strengthening. Stress relaxation was conducted under 0.2% strain for 8 h followed by 40 h recovery in water. The stress‒time relationship is analyzed by nonlinear least-squares data-fitting. The fitted Prony series predicts the sample’s history under monotonic loading. Results showed that the control failed after the first loading‒unloading‒recovery cycle with permanent deformation. While for the experimental sample, the displacement was almost completely recovered and the Young’s modulus increased significantly after the first test cycle. The experimental polymer exhibited higher degree of conversion, lower leachate, and time-dependent stiffening characteristics. The autonomous-strengthening reaction persists in the aqueous environment leading to a network with enhanced resistance to deformation. The results illustrate a rational approach for tuning the viscoelasticity of durable dental adhesives.

## 1. Introduction

Hybrid organic‒inorganic polymers have been explored extensively by both industrial and academic research communities [1,2,3]. These hybrid materials offer several advantages including the ability to tune the properties through composition, ratio, and manufacturing process, such as chemical catalysis, photo-induced reaction, post-curing reactions, solvents, etc. [4,5,6]. As an example, hybrid materials with unique properties have been developed using the photoacid-induced sol‒gel reaction and UV irradiation [7]. This approach combines free-radical polymerization, cationic polymerization, and acid-catalyzed sol‒gel reaction. The inorganic moiety, methoxysilane, was incorporated into the polymer backbone or side chains.

Our group developed hybrid polymers that coupled visible light-induced sol–gel reaction with free radical polymerization. Based on the polymerization kinetics, solubility, and mechanical properties, we proposed the following mechanism for the autonomous strengthening that was recorded for these novel polymers [8,9]. When the liquid resin is irradiated by visible-light, the polymethacrylate-based network is formed by free-radical polymerization of comonomers, for example, 2-hydroxyethyl methacrylate (HEMA) and bisphenol A glycerolate dimethacrylate (BisGMA). Simultaneously, the alkoxysilane groups are hydrolyzed in a reaction catalyzed by the photoacid produced during the visible-light irradiation. Soaking the resin in water or lactic acid furthers the autonomous hydrolysis and condensation of the alkoxysilyl moieties, creating new crosslink points. The resulting silanol groups react with other silanol groups or the hydroxyl groups of HEMA or BisGMA to form covalent bonds [10]. The autonomic sol‒gel reaction continues in the wet environment, leading to intrinsic reinforcement of the network. The autonomous-strengthening adhesive provides enhanced hydrolytic stability, increased mechanical properties, and decreased degradant release [11,12].

The properties of these autonomous strengthening adhesives contrast distinctly with current methacrylate-based adhesives. That is, current methacrylate-based adhesives, aged in water or lactic acid, undergo hydrolytic degradation that leads to decreased mechanical properties and significant degradant release [13]. Bond durability and quality are two of the major challenges for dental adhesives. For example, in spite of the improved clinical outcomes noted with contemporary universal adhesives [14], there are a variety of patient characteristics that threaten the durability of dental adhesives. Patient characteristics include high caries-risk, gingival cavity margins, and posterior teeth [15]. Indeed, most dental resins are degraded by the caustic conditions present in the oral cavity and this activity leads to the long-term release of degradation by-products [16,17]. These by-products accumulate at the resin/tooth interface and increase the virulence of cariogenic bacteria, provoking a degradative positive-feedback loop [16]. This degradative feedback loop is central to the failure of dental adhesives.

Numerous strategies have been proposed to increase the stability and durability of dental adhesives and although a diverse catalogue of properties has been considered, one area that has been largely neglected is viscoelasticity, in other words, time- and rate-dependent behavior of the material. Neglecting this important parameter hampers the development of clinically durable dental adhesives. For example, investigators showed that time- and rate-dependent behavior can have a profound effect on stress concentration at the dentin/adhesive (d/a) interface [18,19,20] and failure at the d/a interface is determined by the component whose stress concentration is closest to its failure strength [19,20,21,22,23].

Materials that exhibit time-dependent mechanical properties, for example, viscoelasticity, stress relaxation, and creep are reminiscent of living systems. The extracellular matrix (ECM) which plays a pivotal role in numerous cell functions exhibits stress relaxation, in other words, time-dependent decrease in resistance to deformation under constant strain. Hydrogels that mimic this behavior are widely used in cell culture studies and it is well established that the mechanical properties of the substrate regulate adherent cell behavior [24]. Indeed, modulating the architecture of hydrogels to achieve stress relaxation that mimics the extracellular matrix is an area of intense investigation [24,25]. In contrast to hydrogel-based materials, there has been limited investigation of stress relaxation in materials that develop durable, highly crosslinked networks with substantial mechanical strength.

In this paper, the effect of network structure on mechanical property evolution was studied in polymethacrylate-based polymers with or without γ-methacryloxypropyl trimethoxysilane (MPS). The dynamic mechanical properties and stress relaxation behavior were measured. The stress‒time relationship in the relaxation test is analyzed by nonlinear least squares data fitting to obtain relaxation times and moduli. The fitted Prony series is used to demonstrate the stiffening behavior by simulating performance under monotonic loading. The simulation provides information that is not directly accessible through the stress–time relationship obtained from the stress relaxation test. The simulation leads to the elastic modulus comparison between the polymer specimens, in other words, polymers with and without autonomous strengthening. To our knowledge, this is one of first examples studying time-dependent mechanical properties, in other words, stress relaxation, in polymers that develop highly crosslinked network structures with substantial mechanical strength.

## 2. Materials and Methods

### 2.1. Materials

**Scheme 1:**
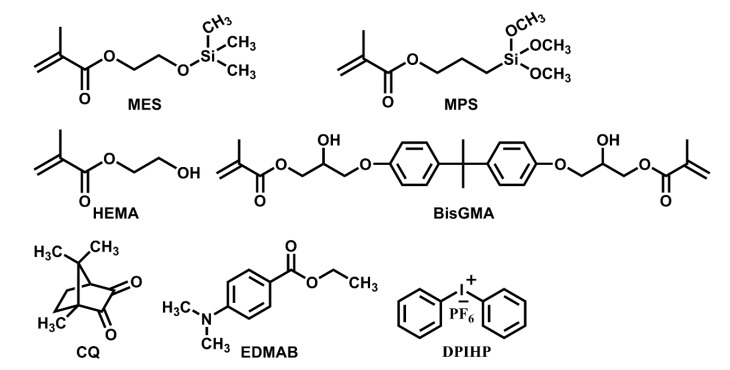
Chemical structures of components used in the formulations.

The following formulation components were obtained from Sigma-Aldrich (St. Louis, MO, USA): Bisphenol A glycerolate dimethacrylate (BisGMA), 2-hydroxyethyl methacrylate (HEMA), camphoroquinone (CQ), ethyl-4-(dimethylamino) benzoate (EDMAB), and diphenyliodonium hexafluorophosphate (DPIHP). The formulation components γ-methacryloxypropyl trimethoxysilane (MPS) and methacryloxyethoxytrimethylsilane (MES, 95%) were obtained from MP Biomedicals (Solon, OH, USA) and Gelest Inc. (Morrisville, PA, USA), respectively. The chemicals were used as received without further purification. Scheme 1 provides the chemical structures of the components in the formulations.

### 2.2. Preparation of Formulations

Neat methacrylate resins (shown in Table 1) were made by mixing 58 wt% HEMA, 30 wt% BisGMA, and 10 wt% MES (C1) or MPS (E1). Three components, for example, CQ (0.5 wt%), EDMAB (0.5 wt%), and DPIHP (1.0 wt%) were used as the three-component photoinitiator (PIs) system [26]. Mixtures of the monomers/PIs were prepared in brown glass vials under amber light [9].

### 2.3. Water Miscibility of Adhesive Formulations

We reported the protocol for determining water miscibility in prior publications [27,28]. The neat resin (about 0.5 g) was weighed in a dark-colored vial and water was added in increments (about 0.005 g/increment) until the mixture became turbid. At this point, the percentage of water in the mixture was noted (*w_1_*). The mixture was then back-titrated using neat resin until the turbidity disappeared. The percentage of water in the non-turbid mixture was noted as w_2_. Three measurements were recorded for each formulation and water miscibility (*W_wm_*, %) was calculated as the average of w_1_ and w_2_.

### 2.4. Real-Time Conversion of C=C Bond

FTIR spectroscopy was used to determine the degree of conversion (DC) [28]. The photopolymerization behavior was monitored in situ at a resolution of 4 cm^−1^ using an infrared spectrometer (Spectrum 400, Perkin-Elmer, Waltham, MA, USA). Spectra were collected continuously throughout the polymerization process using a time-based spectrum collector (Spectrum TimeBase, Perkin-Elmer, Waltham, MA, USA) and three measurements were performed for each formulation. The band ratio profile—1637 cm^−1^ (C=C)/1715 cm^−1^ (C=O) [29] was used to monitor methacrylic double bond conversion. The value for the DC is based on the average of the last 30 values of the time-based spectra.

### 2.5. Preparation of Polymer Specimens

The sample preparation protocol for dynamic mechanical analysis of polymer beams has been reported [26]. In brief, the resins were injected into a glass-tubing mold (Fiber Optical Center, Inc, Part No. ST8100, Bedford, MA, USA) and cured with visible light. Rectangular beam specimens (1 mm × 1 mm and length of 15 mm) were used for dynamic mechanical and stress relaxation test. Disc-shaped specimens (4 mm diameter and 1.2 mm thickness) were used for the water sorption and leachates study. Disc-shaped specimens were prepared in a Tzero Hermetic Lid (P/N: 900797.901 TA Instruments, New Castle, DE, USA), the surface was protected with a Mylar film, and the specimen was light-cured for 40 s.

### 2.6. Water Sorption of Adhesive Polymer

The experimental protocol for the water sorption analyses has been reported [28]. In brief, five disc-shaped specimens were prepared for each polymer formulation. After 1-hour post-processing at 23 ± 2 °C, the samples were immersed in distilled water to prewash for 7 days at 23 ± 2 °C. Next, the specimens were dried in a convection oven at 37 °C for 2 days, and then stored in a vacuum oven in the presence of freshly dried silica gel at 37 °C. The samples were removed every 24 h and weighed using an electronic balance (Mettler Toledo, XS205 Dual range, Columbus, OH, USA). The procedure continued until a constant mass (*m_1_*) was measured. Thus, the process for water sorption involved prewash (7 days), drying and weighing every 24 h until a constant mass was achieved, and finally, the dried specimens were soaked in distilled water. The samples were removed from the water at fixed intervals (3 h, 6 h, 12 h, 24 h, 48 h, and 72 h), blotted to remove excess water, weighed (*m_2_*) and returned to the water until a constant weight was obtained. The values (%) for water sorption (*W_sp_*) were calculated by the following equation:

(1) $$W_{sp}=\frac{m_{2}-m_{1}}{m_{1}}\times 100\%$$

### 2.7. DMA Test and Prony Series Evaluation

**Scheme 2:**
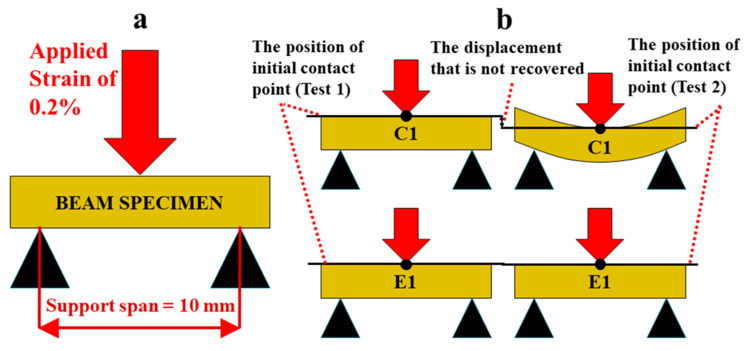
(**a**) Representation of stress relaxation test applied on a polymer beam specimen using a 3-point-bending test stage where the support span is 10 mm and the applied strain is 0.2%, and (**b**) the detection of the non-recovered displacement after the first load‒unload‒recovery cycle that reveals the almost complete recovery of E1 whereas C1 cannot recover.

Stress relaxation test of the rectangle polymer specimens in wet conditions was performed using a TA Instruments Q800 DMA (New Castle, DE, USA) with the 3-point bending submersion clamp [26]. The rectangular beam sample was post-cured at 23 ± 2 °C for 1 h. Following post-curing, the sample was soaked in water at 37° for 24 h. The sample was tested under 0.2% strain at 25 ± 1 °C for 8 h followed by 40 h recovery in water at 37 °C. This stress relaxation and recovery procedure was repeated up to five times unless the sample exhibited permanent deformation. Three specimens (*n* = 3) were tested for each formulation. With this testing modality, the resulting stress which corresponds to the material’s resistance to deformation is measured over time while the specimen is under constant strain (shown in Scheme 2a). In addition to stress‒time data, the position of initial contact point at each test was recorded to determine the amount of recovery under wet conditions (shown Scheme 2b).

The obtained stress versus time data were analyzed by using a Prony-series-fitting approach via MATLAB platform (Mathworks, Natick, MA, USA) [12], to find the relaxation times and corresponding moduli. It is noted that the evolution of mechanical performance across different time scales cannot be simply accessed using the stress vs. time relationship obtained from stress relaxation tests. To this end, the generalized Maxwell model was used to model material constitutive behavior where it is represented by Prony series [30,31]. Furthermore, the obtained Prony parameters were incorporated into the simulation of mechanical behavior under monotonic loading in order to evaluate the mechanical performance.

DMA temperature ramp tests were performed on separate rectangular beam specimens using a TA instruments Q800 DMA (TA Instruments, New Castle, USA) with a 3-point bending clamp [8]. Three specimens were tested for each formulation. The following parameters were assigned for temperature ramp testing: displacement amplitude of 15 µm, frequency of 1 Hz, and preload force of 0.001 N [32]. The temperature was ramped at the rate of 3 °C/min from 20 to 180 °C. The testing parameters of the second scan were the same as the first scan. Glass transition temperature (T_g_) is determined as the position of the maximum peak on tan δ versus temperature measured by DMA [33]. In addition, to determine the rubbery modulus and T_g_ of soaked sample in dry conditions, the beam specimens were soaked in water at 37 °C for 1, 3, and 5-days, respectively. The soaking times corresponded to the time scale for the 1st, 2nd, and 3rd stress relaxation cycles. The hydrated specimens were dried in a vacuum oven in the presence of freshly dried silica gel at 37 °C and the mechanical properties of the dried specimens were determined using the method described above. The inverse ratio of the rubbery modulus to temperature was used to calculate the relative crosslink density of the polymers [26,34].

### 2.8. Leachable HEMA Study by High Performance Liquid Chromatography (HPLC)

The disc-shaped samples used for the leachate study, HEMA, were submerged in 1.5 mL water at 37 ± 0.5 °C for 1–9 days. The 0.5 mL extracts were removed at various time intervals, namely, 1, 3, 5, and 9 days, and analyzed for HEMA concentration. After each collection, fresh water (0.5 mL) was added to the vial. The extracts were analyzed using high-performance liquid chromatography (HPLC, Shimadzur LC-2010C HT, software EZstart, version 7.4 SP2). The system was equipped with a 250 × 4.6 mm column packed with 5 µm C-18 silica (Luna, Phenomenex Inc., Torrance, CA, USA) and the mobile phase was acetonitrile/water (50/50, *v*/*v*). The operating conditions were as follows: 0.5 mL/min flow rate, detection at 208 nm, 20 µL sampling loop, and column temperature 40 °C. The system was calibrated using HEMA at concentrations of 5, 10, 20, 50, 100, 250, and 500 mg/L in water. The concentration of HEMA in the extracts was determined using the calibration curve (linear fitting of HEMA (5¨C100 mg/L, R^2^ = 0.997). The concentration calculation was based on the intensity of the chromatographic peaks at the corresponding retention time (HEMA 7.0 min). The HPLC analysis was performed using the extract of five samples from each formulation.

### 2.9. Statistical Analysis

The results from the following experiments: water miscibility, water sorption, degree of conversion (FTIR), and accumulative concentration of HEMA leachate (HPLC) were analyzed using one-way analysis of variance (ANOVA) together with Tukey’s test at α = 0.05 (Microcal Origin Version 8.0, Microcal Software Inc., Northampton, MA, USA). The statistical analysis was used to identify significant differences in the means.

## 3. Results

**Figure 1:**
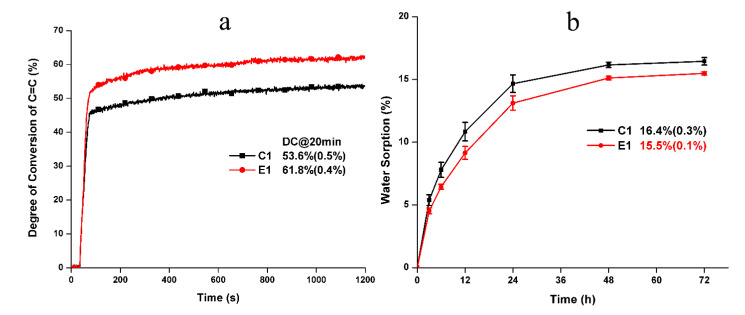
Real-time degree of conversion of C=C bond (**a**) and the water sorption (**b**) of the control and experimental formulations.

The water miscibility values of the control and experimental formulations are 15.6 ± 0.5 wt% and 15.5 ± 0.1 wt%, respectively. There is no statistically significant difference between the formulations (*p* > 0.05). Figure 1a shows the real-time photopolymerization profiles of the control and experimental resin formulations. The experimental resin exhibits a significantly higher degree of conversion (DC) than that of the control (*p* < 0.05). Figure 1b shows the water sorption behavior of the control and experimental formulations. The water sorption of the solid polymers reached a plateau after three days soaking. The final water sorption of C1 and E1 are 16.4 ± 0.3% and 15.5 ± 0.1%, respectively. There is no statistically significant difference in the final water sorption between the formulations (*p* > 0.05).

**Figure 2:**
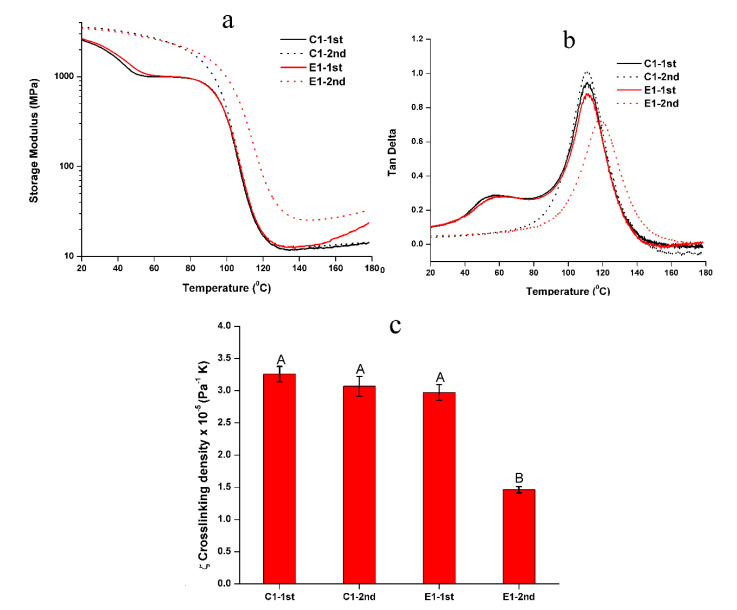
Representative (**a**) storage modulus and (**b**) tan δ vs. temperature of the non-treated C1 and E1 in two-cycle DMA test, and (**c**) the calculated relative crosslinking density (the same letter indicates no statistically significant difference at the 0.05 level).

The representative mechanical behavior of the non-treated C1 and E1 specimens tested in two-cycle temperature ramp is shown in Figure 2 and the summary of rubbery modulus and T_g_ are listed in Table 2. In the 1st cycle, two transition temperatures can be observed for C1 and E1, and the rubbery moduli of C1 and E1 are comparable at about 12–13 MPa. For C1, the first transition temperature and T_g_ are 57.2 ± 0.7 °C and 111.8 ± 0.6 °C, respectively. For E1, the first transition temperature and T_g_ are 60.6 ± 0.7 °C and 111.2 ± 0.5 °C, respectively. In the 2nd test cycle, the first transition peak disappeared. The rubbery modulus of C1 increased from 11.9 MPa to 12.8 MPa, while E1 increased from 13.1 MPa to 26.7 MPa. The T_g_ for C1 and E1 are 112.3 ± 0.7 °C and 118.9 ± 0.3 °C, respectively. The Tg values of C1 are comparable in both cycles (*p* < 0.05). The Tg of E1 in the 2nd cycle is significantly higher than that in the 1st cycle (*p* < 0.05). The calculated relative crosslinking density (ζ) [26,34] of E1 in the 2nd cycle was significantly higher than the C1 and E1 in the 1st cycle (*p* < 0.05).

**Figure 3:**
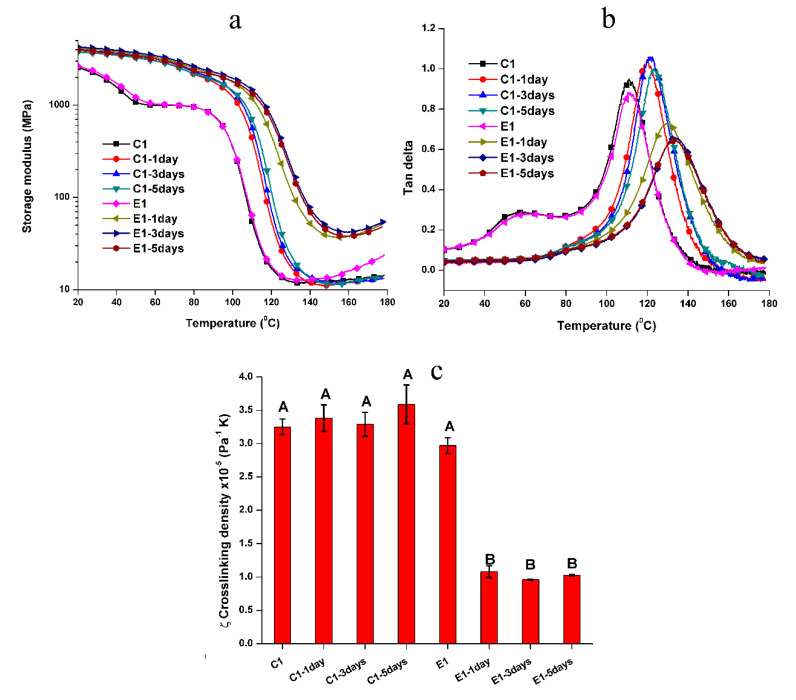
Representative (**a**) storage modulus and (**b**) tan δ vs. temperature, and (**c**) the crosslinking density of the non-treated and treated C1 and E1 in dry conditions (the same letter indicates no statistically significant difference at the 0.05 level).

As noted in Figure 3, there are slight differences in the mechanical properties of the treated samples and non-treated samples in the two-cycle test. Compared to the non-treated C1 and E1, the first transition peak of treated samples disappeared. For the C1 samples, the T_g_ and rubbery moduli are comparable for samples soaked in water for 1, 3, or 5-days. However, for the E1 samples, the T_g_ and rubbery modulus of E1 soaked for 1-day showed a significant increase as compared to the non-treated E1. The relative crosslinking densities, ζ, of the treated E1 specimens were significantly higher than the non-treated/treated C1 and non-treated E1 at the 0.05 level. Meanwhile, the storage modulus profiles of the treated E1 specimens increased gradually with temperature in the rubbery region. These results indicated that the autonomic sol‒gel reaction had not reached a plateau even after 5 days soaking in water.

**Figure 4:**
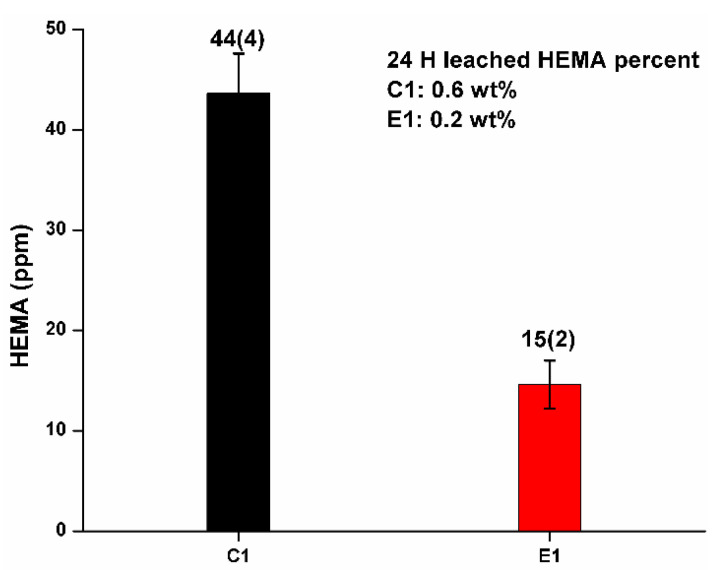
Cumulative HEMA release from the C1 and E1 polymers in water at 37 °C for 24 h.

Figure 4 shows the results of 24 h cumulative HEMA released from the C1 and E1 polymers soaked in water at 37 °C. The cumulative concentrations of HEMA for C1 and E1 was 44 ± 4 ppm and 15 ± 2 ppm, respectively. The percentages of leached HEMA for C1 and E1 were 0.6 wt% and 0.2 wt%, respectively. BisGMA, MES, and MPS were not detected in the elution. Extending the soaking time to 3, 5, and 9-days did not lead to a significant increase in cumulative HEMA release (unpublished results).

**Figure 5:**
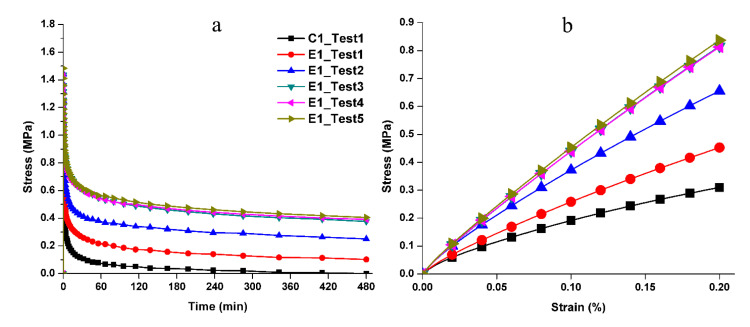
(**a**) Stress‒time relationship from stress relaxation study and (**b**) stress‒strain relationship predicted using Prony-series-fitted parameters.

Figure 5a gives the measured stress‒relaxation behavior. We can observe that control specimen C1 relaxed to zero stress after 480 min indicating its inability to sustain the initial loading (1st loading event). In contrast, E1 undergoes a viscoelastic deformation as shown by the residual stress at the end of 480 min. Moreover, for subsequent loading‒unloading cycles E1 shows a clear trend of increasing residual stress at the end of relaxation. Additionally, we noted that C1 did not recover the applied displacement after the first loading‒unloading‒recovery cycle (Scheme 2b), whereas E1 significantly recovered the applied displacement, that is approximately 31 µm of 35 µm, after loading‒unloading‒recovery cycle. The simulated monotonic loading response obtained using the fitted Prony-series predicts that E1 becomes stiffer after the first and the second testing (Figure 5b). Although after the third and fourth testing, the behavior shows small change. The initial stiffness (during 1st loading event) of E1 is found to be ~203 MPa. The stiffness subsequently increases to ~294 MPa, ~385 MPa, and ~390 MPa for the 2nd, 3rd and later loading cycles.

## 4. Discussion

Degradation of the dentin/adhesive (d/a) interface is a complex process that involves mechanical fatigue as well as chemical/enzymatic degradation of the adhesive and collagen [35,36,37,38]. It is widely accepted that degradation of the d/a interface occurs after photo-curing of the dental adhesive [39,40,41]. Leaching and hydrolysis of the adhesive play a critical role in the degradation process and the effects of these phenomena have been widely investigated [28,37,42,43]. Meanwhile, the effect of physical-mechanical loading on the durability of the d/a interface has not been fully elucidated. Masticatory forces contribute to gap formation and marginal leakage at the d/a interface [44] and these negative effects could be attributed, in part, to plastic deformation of the adhesive [19,45].

### 4.1. Role of Heating

To capture the effect of the autonomous-strengthening reaction on the mechanical properties, two-cycle DMA for non-treated samples, in other words, samples under dry conditions, was conducted. As shown in Figure 2, in the 1st cycle, two transition temperatures were observed. The 1st transition temperature was attributed to the lower conversion in the early stage (1 h post-curing). The unpolymerized components, such as HEMA and the pendant C=C bond of BisGMA, can act as a plasticizer, which contributes significantly to the chain mobility. This transition temperature disappeared in the 2nd cycle. This difference is attributed to polymerization of the unreacted C=C bonds due to the heating. For the non-treated C1, the rubbery moduli and T_g_ values in both cycles were comparable (*p* < 0.05). This result indicated that the major unpolymerized components after light-irradiation were monomethacrylate, namely, HEMA or MES, whose polymeric chains were linear structures and would not contribute to the crosslinked network. In comparison, for the non-treated E1, the rubbery modulus increased from 13.1 MPa in the 1st cycle to 26.7 MPa in 2nd cycle, and the T_g_ increased from 111.5 °C to 118.8 °C. These results indicate higher crosslink density in E1 samples. The results indicate further crosslinking as a result of the sol‒gel reaction during heating [8,9].

### 4.2. Role of Water

We reported that the self-strengthening reaction is accelerated not only by heating but also by water [8,9,11]. The rubbery moduli and T_g_ for the treated E1 increased significantly after one day of aqueous aging. This result indicated that the autonomous-strengthening reaction was promoted by the increased mobility of methoxysilyl functional groups as a result of the polymer network swelling. This trend did not, however, show a linear relationship with soaking time.

In contrast, the rubbery moduli of non-treated and treated C1 samples were comparable (*p* < 0.05). This result indicated that the network structure of the C1 polymer was independent of the post-processing.

The T_g_ of C1 increased significantly from 111.8 °C (non-treated sample) to about 120–122 °C (treated sample). This phenomenon was attributed to the plasticizing effect of unpolymerized components. For the non-treated sample, the unpolymerized components, HEMA and MES, could act as plasticizers during the 1st cycle DMA test. For the treated sample, the majority of unpolymerized HEMA was leached during soaking (as seen in Figure 4). Therefore, the plasticizing effect from the unpolymerized components has been eliminated in the treated C1 samples. The T_g_ of the treated C1 shifted to a higher temperature. The rubbery moduli and T_g_ values were independent of the soaking time which indicated that the network structure of C1 has reached equilibrium after soaking in water for one day.

The inverse ratio (ζ) of modulus in the rubbery region to the absolute T_g_ temperature has been used to present the relative crosslinking density of crosslinked polymers [46]. The ζ values of the non-treated and treated C1 samples were comparable (*p >* 0.05). In comparison, the ζ values of the treated E1 samples were significantly lower (*p* < 0.05) than that of the C1 and non-treated E1. The decreased ζ value indicated the higher crosslinking density for the treated E1 polymer, which was attributed to the further crosslinking as a result of the sol–gel reaction during soaking.

### 4.3. Leaching Behavior of Aged Polymer Samples

The cumulative HEMA concentration for C1 and E1 was 44 ± 4 ppm and 15 ± 2 ppm after one day, respectively. The corresponding percent of leached HEMA was 0.6 wt% and 0.2 wt%, respectively. The increase in soaking time from 3 to 9 days did not result in a significant increase in the concentration of HEMA leached from the samples. Compared to our previous investigation that used ethanol as the aging solvent, the amount of leached HEMA was significantly lower and no BisGMA was detected [8,9,11,47].

### 4.4. Network Structure Response to Cyclic Loading

Although studies have shown improved properties for dental adhesives that achieve autonomous strengthening [8,9,11,12], little is known about the effect of this reaction on the cyclic-loading response during the early stages of network structure development. Within the clinical environment, the d/a interface will be subjected to cyclic stress and these stresses challenge both the durability of the adhesive and the integrity of the d/a interface [31,48]. In this study, we used a process of cyclic loading‒unloading‒recovery to mimic conditions that occur in the mouth. In this testing modality, a constant strain was applied and the resulting stress which corresponds to the material’s resistance to deformation was measured over time. Specifically, the samples were tested for 8 h under 0.2% strain (25 ± 1 °C) followed by 40 h recovery in water at 37 °C.

The stress relaxation results with E1 clearly show that the mechanical properties improved over time via the autonomous-strengthening reaction (Figure 5a). These observations were assisted by the predictions from the Prony-series-analysis where the Young’s modulus for E1 increases over the course of five loading‒unloading‒recovery cycles. This trend clearly reveals the benefit of the autonomous-strengthening phenomenon. E1 showed clear resistance to deformation under those conditions that led to failure in C1 samples. These results indicated that the network structure and mechanical behavior depended significantly on the autonomous strengthening reaction in wet conditions. Therefore, the null hypothesis that the mechanical properties of polymer specimens are independent of the autonomous-strengthening reaction was overruled.

**Scheme 3:**
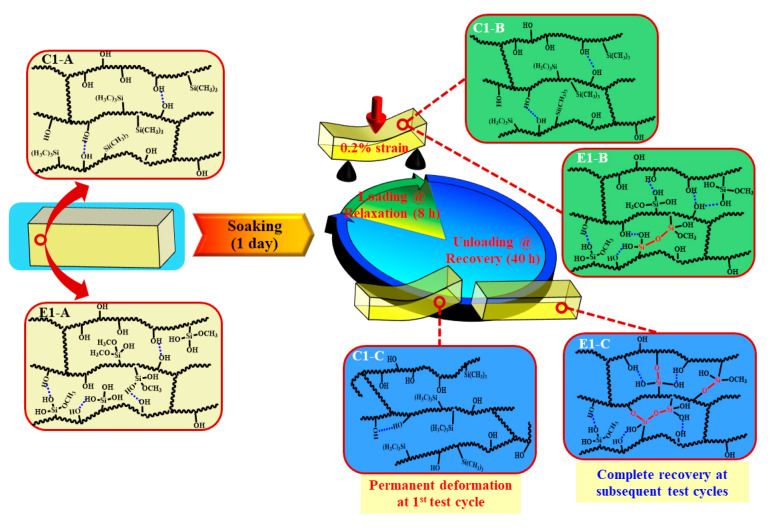
Schematic illustration of evolution of the polymethacrylate-based network structures during the recurring stress relaxation test: (C1/E1-A) C1/E1 network structures after soaking in water for 1 day at 37 °C; (C1/E1-B) C1/E1 network structures at the stress loading and relaxation; (C1/E1-C’) C1/E1 network structures during the recovery period. (Blue dash line presents the hydrogen bonds between OH•••OH or Si―OH•••OH; red solid line presents the new formed covalent bond).

We are proposing the network structure evolution based on the stress relaxation test and DMA results as shown in Scheme 3. These results are obtained collectively from the control and experimental samples at the 1st loading-unloading-recovery cycle. From the DMA results of the non-treated specimens (see Table 2 and Figure 2), it is clear that the initial crosslinking densities of the non-treated C1 and E1 (experienced 1 h post-curing at 25 ± 2 °C) are comparable. These results are attributed to the polymethacrylate-based crosslinked network structures that are formed through free-radical polymerization during light irradiation. The sol‒gel reaction was very limited in the E1 sample after photocuring and 1 h post-curing [9]. After the C1 and E1 samples are aged in water for one1 day, the unpolymerized HEMA has been leached from the polymers (HPLC results, Figure 4) and the network structure has almost reached an equilibrium swelling status (water sorption results, Figure 1b). The effect of the trapped free radical species [49] on the crosslinking of the network structure is negligible. In contrast, the sol–gel reaction was promoted by soaking the E1 polymer in water for one day and this activity led to a significant increase in crosslink density in the E1 polymer as compared to C1.

The polymethacrylate-based matrix, shown as Scheme 3 C1/E1-A, provides covalent bond crosslinking through BisGMA but also hydrogen-bonds (OH•••OH) [50]. As for C1, MES lacks hydrogen bond donor/acceptor functionality and the formation of hydrogen bonds is mainly attributed to the hydroxyl groups in the side chains (polyHEMA). In comparison, for E1, in aqueous solution, the methoxysilane group in MPS readily hydrolyzed and the formed silanol groups (Si―OH) interacted with hydroxyl groups in the side chains. As a result, mobility of the backbone chain is expected to be suppressed by the hydrogen bonds among the Si―OH•••OH and Si―OH•••HO―Si (Scheme 3 E1-A/B).

Although hydrogen bonding between the side chains is a weak interaction, it still plays an important role in tuning the chain mobility and flexibility of the polymeric chains. Due to the inherent viscoelasticity of polymer materials, the plastic flow of the polymer chains is an essential part of the stress response during the loading cycle. As for C1, the hydrogen bonds between the OH•••OH could be broken by local tensile forces and relaxation of the polymer chains occurred quickly. The hydrogen bonds cannot reform spontaneously and permanent deformation will be observed (Scheme 3 C1-B/C). For E1, silanol groups formed through the hydrolysis of methoxysilane in wet conditions—the amount of hydrogen bond donor/acceptor is theoretically 27% larger than that in C1 (based on the amount of hydroxy group from HEMA). Meanwhile, the covalent bonds could be gradually formed through the condensation reactions between silanol groups, silanol-hydroxyl groups, or both [10,51,52], which further decreases the mobility of the polymer chain, hinders the plastic flow, and finally improves the deformation resistance. Within the recovery period, covalent bonds are formed through the condensation reactions. This phenomenon was supported by the gradually increased stiffness observed in the loading–unloading–recovery cycles (Figure 5).

In summary, due to the inherent viscoelastic characteristic of polymers, under a constant strain, rearrangement of the side chain or flexible chains occurs and the stress undergoes relaxation. For the control, the network structure almost reached equilibrium during the first day of aging (see Figure 1b) and very limited crosslinking reaction occurred (see Table 2). Leaching of the non-crosslinked chains and the rotation or rearrangement of the side groups within the C1 network resulted in permanent deformation. In comparison, for the experimental polymer, higher crosslinking density was promoted by the sol‒gel reaction during the first day of aging—this led to a reduction in the non-crosslinked chains and chain mobility. The viscous behavior of the polymer was, thus, retarded. Therefore, after the first loading–unloading–recovery cycle, ~90% of the applied displacement was recovered. This result indicates that the autonomous-strengthening reaction propagates over an extended time period and the generated hybrid network structure has enhanced resistance to deformation.

The current investigation focused on relatively stiff polymers which inhibited the analysis of the evolving network structure during light irradiation. Rheological property investigations with hydrogel-like systems could be used to advance our understanding of hybrid materials that achieve autonomous strengthening [53,54,55]. In addition, future work should explore the effect of the organic phase on crosslinking density and flexibility of polymer chain. Additional processing parameters such as irradiation intensity and time, pH, and aging solvent should be investigated. The results from these investigations will likely identify parameters that must be exploited to optimize the autonomous strengthening dental adhesive.

## 5. Conclusions

The time-dependent mechanical properties of adhesive with autonomous strengthening capabilities have been investigated. The network structure and mechanical behavior of the hybrid adhesive system were significantly dependent on the autonomous strengthening reaction under wet conditions. The stress relaxation test and DMA results suggested that the network structure evolved during aging in water. The stress–time relationship was post-processed via Prony-series-analysis and the stiffening behavior was captured in the simulation. Overall, the designed hybrid network structure offered enhanced deformation resistance over an extended period of time as the autonomous-strengthening reaction propagated. The benefits of this hybrid adhesive system which include higher degree of conversion, lower leachate, and time-dependent stiffening characteristics, point towards a new strategy for durable dental adhesives. Enhanced durability translates to increased resistance to degradation and failure. This strategy could be introduced into soft biomaterials to tune the mechanical properties (viscoelasticity) for a range of applications including tissue engineering, wound dressings, and scaffold materials.

## Figures and Tables

**Table 1:** Composition of the control and experimental formulations.

Component (wt%)	C1	E1
HEMA	58	58
BisGMA	30	30
MES	10	-
MPS	-	10
CQ	0.5	0.5
EDMAB	0.5	0.5
DPIHP	1.0	1.0

**Table 2:** Rubbery modulus, T_g_, and crosslinking density of the C1 and E1 specimens.

Type	Sample	No.	Rubbery Modulus (MPa)	T_g_ (°C)	ζ (× 10^–5^ Pa^–1^ K)
Non-treated	C1	1st	11.9^A^ ± 0.5	111.8^A^ ± 0.6	3.26^A^ ± 0.12
		2nd	12.8^A^ ± 0.3	112.3^A^ ± 0.8	3.07^A^ ± 0.15
	E1	1st	13.1^A^ ± 0.6	111.2^A^ ± 0.5	2.97^A^ ± 0.12
		2nd	26.7^B^ ± 1.2	118.9^B^ ± 0.3	1.46^B^ ± 0.05
Treated	C1	1 day	11.8^A^ ± 0.5	120.2^B^ ± 0.3	3.38^A^ ± 0.20
		3 days	12.0^A^ ± 0.6	122.2^B^ ± 0.7	3.29^A^ ± 0.18
		5 days	11.1^A^ ± 0.9	123.2^B^ ± 0.1	3.59^A^ ± 0.29
	E1	1 day	37.4^B^ ± 3.3	130.4^B^ ± 1.9	1.08^B^ ± 0.09
		3 days	42.6^B^ ± 0.7	133.9^B^ ± 0.8	0.96^B^ ± 0.01
		5 days	39.5^B^ ± 0.4	133.1^B^ ± 0.5	1.03^B^ ± 0.01

Means followed by the same letter indicate no statistically significant difference from the control (C1-1st) at the 0.05 level. Non-treated means the polymer beam only experienced 1 h post-curing at 23 ± 2 °C and was used for DMA test. Treated indicates the polymer beam experienced 1 h post-curing at 23 ± 2 °C, then was soaked in water at 37 °C for 1, 3, or 5 days, and completely dried for DMA test.
